# Cancer Stem Cells in Metastatic Head and Neck Cutaneous Squamous Cell Carcinoma Express Components of the Renin-Angiotensin System

**DOI:** 10.3390/cells10020243

**Published:** 2021-01-27

**Authors:** Sam Siljee, Olivia Buchanan, Helen D. Brasch, Nicholas Bockett, Josie Patel, Erin Paterson, Gordon L. Purdie, Paul F. Davis, Tinte Itinteang, Swee T. Tan

**Affiliations:** 1 Gillies McIndoe Research Institute, Wellington 6242, New Zealand; sam.siljee@gmri.org.nz (S.S.); olivia.buchanan82@hotmail.com (O.B.); hdquinn@fastmail.fm (H.D.B.); nick.bockett@gmri.org.nz (N.B.); josie.patel@gmri.org.nz (J.P.); erin.paterson@gmri.org.nz (E.P.); gordon.purdie@otago.ac.nz (G.L.P.); paul.davis@gmri.org.nz (P.F.D.); tinte01@yahoo.com (T.I.); 2 Biostatistical Group, Dean’s Department, University of Otago, Wellington 6021, New Zealand; 3 Wellington Regional Plastic, Maxillofacial and Burns Unit, Hutt Hospital, Lower Hutt 5010, New Zealand; 4 Department of Surgery, The Royal Melbourne Hospital, The University of Melbourne, Melbourne, VIC 3050, Australia

**Keywords:** cutaneous squamous cell carcinoma, cancer stem cells, angiotensinogen, renin, prorenin receptor, angiotensin-converting enzyme, angiotensin-converting enzyme 2, angiotensin II receptor 1, angiotensin II receptor 2

## Abstract

We investigated the expression of components of the renin-angiotensin system (RAS) by cancer stem cell (CSC) subpopulations in metastatic head and neck cutaneous squamous cell carcinoma (mHNcSCC). Immunohistochemical staining demonstrated expression of prorenin receptor (PRR), angiotensin-converting enzyme (ACE), and angiotensin II receptor 2 (AT_2_R) in all cases and angiotensinogen in 14 cases; however, renin and ACE2 were not detected in any of the 20 mHNcSCC tissue samples. Western blotting showed protein expression of angiotensinogen in all six mHNcSCC tissue samples, but in none of the four mHNcSCC-derived primary cell lines, while PRR was detected in the four cell lines only. RT-qPCR confirmed transcripts of angiotensinogen, PRR, ACE, and angiotensin II receptor 1 (AT_1_R), but not renin or AT_2_R in all four mHNcSCC tissue samples and all four mHNcSCC-derived primary cell lines, while ACE2 was expressed in the tissue samples only. Double immunohistochemical staining on two of the mHNcSCC tissue samples showed expression of angiotensinogen by the SOX2+ CSCs within the tumor nests (TNs), and immunofluorescence showed expression of PRR and AT_2_R by the SOX2+ CSCs within the TNs and the peritumoral stroma (PTS). ACE was expressed on the endothelium of the tumor microvessels within the PTS. We demonstrated expression of angiotensinogen by CSCs within the TNs, PRR, and AT_2_R by the CSCs within the TNs and the PTS, in addition to ACE on the endothelium of tumor microvessels in mHNcSCC.

## 1. Introduction

There is a high incidence of skin cancer in New Zealand and Australia [1] due to high sun exposure among a susceptible, fair-skinned Caucasian population [2]. Squamous cell carcinoma (SCC) is the second most common form of skin cancer [3], with 60% occurring in the head and neck [4]. About 2.5% of cutaneous SCC develop metastasis, most commonly to the regional lymph nodes [2].

Cancer cells with stem-cell properties—i.e., the ability to self-renew, proliferate, and differentiate into other cell types—have been termed “cancer stem cells” (CSCs) [5], and are the proposed origin of cancer [6]. It is assumed that CSCs result from mutations in normal resident stem cells or progenitor cells, and contribute to tumor progression and metastasis [7]. They can generate all cell types within a tumor and differentiated progeny which form the bulk of a tumor [5], and are responsible for the growth, development, and acquisition of a metastatic phenotype [8]. They have also been attributed to cancer relapse through repopulation, following resistance to conventional treatment such as chemotherapy and radiotherapy [7].

CSCs can be identified by their expression of embryonic stem cell (ESC) markers [9] and have been demonstrated in many cancer types [7], including oral cavity SCC (OCSCC) of different subsites [10,11,12,13], glioblastoma [8], renal clear cell carcinoma [14], primary [15] and metastatic [16] colon adenocarcinoma, and metastatic malignant melanoma to regional lymph nodes [17] and the brain [18]. We recently showed the presence of CSC subpopulations within primary head and neck cutaneous SCC (HNcSCC) [19] and metastatic HNcSCC (mHNcSCC) [20]. These CSC subpopulations within primary HNcSCC and mHNcSCC express ESC markers OCT4, SOX2, KLF4, NANOG and c-MYC [9,10], which are involved in the induction of pluripotent stem cells [9].

The renin-angiotensin system (RAS) is classically associated with cardiovascular homeostasis-regulation of blood pressure and blood volume. Renin binds to the prorenin receptor (PRR) and converts angiotensinogen to angiotensin I (ATI). Angiotensin-converting enzyme (ACE) then converts ATI to angiotensin II (ATII), which acts on the ATII receptor 1 (AT_1_R) or ATII receptor 2 (AT_2_R) to produce physiological effects [21]. Recently, another form of ACE, i.e., ACE2, was discovered, which has a distinct role in cleaving ATII to form the vasodilatory heptapeptide angiotensin 1-7 [AT(1-7)] [22].

There is increasing evidence for a critical role of the RAS in many pathological states, including the development and progression of cancer [23,24]. Patients administered RAS modulators have a reduced risk of recurrence and death from their cancer [23,25,26,27]. It has also been suggested that modulation of the RAS can influence the fate of hemangioblasts, and thus, that this plays a role in the early stages of differentiation of stem cells [28]. The role of angiotensinogen has not been clearly characterized in cancer; however, genetic polymorphisms are associated with gastric [29] and lung [30] cancer, but not OCSCC [31]. Overexpression of angiotensinogen reduces angiogenesis and tumor growth in a hepatocellular carcinoma mouse model [32] and reduces breast cancer proliferation and metastasis [33]. This suggests that the observed effects of heightened RAS on malignancy may be due to downstream signaling via ATII, as opposed to direct effects of angiotensinogen. Renin, acting through PRR, is known to be involved in Wnt/β-catenin signaling, which, in turn, has been implicated in carcinogenesis [24,34]. ACE inhibitors have been shown to reduce tumor angiogenesis, likely mediated through vascular endothelial growth factor (VEGF) [35,36]. This has also been demonstrated in HNcSCC [37]. Furthermore, ACE gene polymorphisms that confer reduced ACE activity are also associated with reduced cancer occurrence [38,39], although other studies have found no association [40]. The ACE2/AT(1-7)/Mas receptor axis has been shown to be anti-angiogenic, antimetastatic, and to direct cell differentiation, including reducing endothelial-to-mesenchymal transition (EMT) [23,41,42]. AT_1_R expression has been linked to differentiation and cancer progression [43], with its overexpression being associated with more aggressive tumors, as it appears to be upregulated during the progression to malignancy [23,44]. AT_2_R provides protection against hypoxia, limits inflammation, and promotes healing [45]. In conjunction with antiproliferative and anti-angiogenic functions [46], AT_2_R is thought to have a protective effect against cancer [23].

We have recently demonstrated the expression of components of the RAS: PRR, ACE, AT_1_R, and AT_2_R by the CSCs in different cancer types, including OCSCC of different subsites [47,48], glioblastoma [8], metastatic malignant melanoma to the brain [18] and regional nodes [49], metastatic colon adenocarcinoma [50], and primary HNcSCC [51]. This study aimed to investigate the expression of components of the RAS: angiotensinogen, renin, PRR, ACE, ACE2, AT_1_R, and AT_2_R, in relation to the CSC subpopulations we have identified in mHNcSCC [20].

## 2. Materials and Methods

### 2.1. mHNcSCC Tissue Samples

mHNcSCC tissue samples from 20 patients aged 51–87 (mean, 77.7) years (Table S1), including those used in our previous study [20], were sourced from the Gillies McIndoe Research Institute Tissue Bank. This study was approved by the Central Regional Health and Disability Ethics Committee (Ref. 12/CEN/74) with written informed consent from all patients.

### 2.2. mHNcSCC-Derived Primary Cell Lines

Primary cell lines were derived from available fresh surgically excised mHNcSCC tissue samples from four patients from the original cohort of 20 patients. Small tissue pieces were embedded in layers of Matrigel (cat#356234, Corning, Tewsksbury, MA, USA), subsequently extracted following abundant growth using Dispase (cat#354235, Corning), and transferred to adherent culture flasks. Cells were explanted, cultured, and passaged in DMEM media (cat#10569010, Gibco, Rockford, IL, USA), supplemented with 10% fetal calf serum (cat#10091148, Gibco), 5% mTeSR^TM^ (cat#85850, StemCell Technologies, Vancouver, BC, Canada), 1% penicillin-streptomycin (cat#15140122, Gibco), and 0.2% gentamicin/amphotericin (cat#R01510, Gibco). All cultures were incubated at 37 °C under an atmosphere of 5% CO_2_. All primary cell lines used for the experiment were between passages 8 and 10.

### 2.3. Histochemical and Immunohistochemical Staining

Hematoxylin and eosin (H&E) staining was performed on 4 μm thick formalin-fixed paraffin embedded sections of tumor tissue from all patients, to confirm the presence of mHNcSCC by an anatomical pathologist. Immunohistochemical staining was performed on these sections using primary antibodies for angiotensinogen (1:50; cat#79299S, Cell Signaling, Danvers, MA, USA), renin (1:500; cat#14291-1-AP, Proteintech, Rosemont, IL, USA), PRR (1:500; cat#ab40790, Abcam, Cambridge, MA, USA), ACE (1:50; cat#PA5-83080, Invitrogen, Carlsbad, CA, USA), ACE2 (1:1000; cat#MAB933, R&D Systems, Minneapolis, MN, USA), and AT_2_R (1:2000; cat#NPBI-77368, Novus Biologicals, Littleton, CO, USA), with 3,3′-diaminobenzidine (ready-to-use; cat#DS9800, Leica, Wetzlar, Germany) as the chromogen.

All antibodies were diluted with BOND primary antibody diluent (cat#AR9352, Leica). Dako Mounting Medium (cat#CS703, Dako, Glostrup, Denmark) was used to mount slides. Negative controls for immunohistochemical staining were either run with rabbit (ready-to-use; cat#IR600, Dako) or mouse (ready-to-use; cat#IR750, Dako) isotype controls, depending on the primary antibody used.

To investigate the localization of components of the RAS in relation to CSCs or tumor microvessels, double immunohistochemical or immunofluorescence co-staining was performed with either an ESC marker or endothelial marker on two mHNcSCC tissue samples from the original cohort. Double immunohistochemical staining (ready-to-use; cat#DS9800, Leica) was run as for immunohistochemical staining above with the exception that the post-primary antibody was replaced with rabbit anti-rat IgG (1:100; cat#312-005-045, Jackson ImmunoResearch, West Grove, PA, USA) to detect SOX2 (1:100; cat#14-9811-82, Invitrogen). Red detection was then performed immediately after with the BOND Polymer Refine Red Detection kit (ready-to-use; cat# DS9390, Leica) to detect angiotensinogen. Immunofluorescence co-staining of PRR and AT_2_R was performed with primary antibodies against ESC marker SOX2 (1:100; cat#14-9811-82, Invitrogen), with ACE co-staining with the endothelial marker CD31 (ready-to-use; cat#PA0414, Leica). For primary antibody detection, secondary antibodies or amplification kits were used: Alexa Fluor anti-mouse 488 (1:500; cat#A-21202, Invitrogen), Alexa Fluor antirabbit 594 (1:500; cat#A-21207, Invitrogen), Alexa Fluor antirat 647 (1:500; cat#A-21247, Invitrogen), or Alexa Fluor antirabbit 594 (ready-to-use; cat#DK-1594, Vector Laboratories, Burlingame, CA, USA). Slides were mounted in Vectashield HardSet antifade mounting medium and counter-stained with 4′6-diamino-2-phenylinodole (cat#H-1500, Vector Laboratories). The angiotensinogen antibody was not optimized for immunofluorescence staining; therefore, double immunohistochemical staining with SOX2 was used. The same primary antibodies and concentrations were used as listed above, with 3,3′-diaminobenzidine as the chromogen alongside the BOND Polymer Refine Red Detection kit (ready-to-use, cat#DS9390, Leica).

Human tissues used for positive controls were liver for angiotensinogen, kidney for renin, ACE, ACE2, and AT_2_R, and placenta for PRR. To determine the specificity of the amplification cascade used in immunofluorescence staining, isotype matched mouse (ready-to-use; cat#IR750, Dako), rabbit (ready-to-use; cat#IR600, Dako), and rat (1:100; cat#14-4321-85, Invitrogen) controls were used as appropriate negative controls. Tissue negative controls confirmed the specificity of primary antibodies. We were unable to validate a suitable antibody for AT_1_R and have therefore excluded this marker from protein-level analysis [52,53,54,55].

### 2.4. Image Capture and Analysis

Immunohistochemical-stained slides were imaged on an Olympus BX53 light microscope fitted with an Olympus SC100 digital camera (Olympus, Tokyo, Japan) and processed with cellSens 2.0 software (Olympus). Immunofluorescence-stained slides were imaged with an Olympus FV1200 biological confocal laser-scanning microscope and processed with cellSens Dimension 1.11 software (Olympus).

### 2.5. RT-qPCR

Total RNA was isolated from four available snap-frozen mHNcSCC tissue samples and four mHNcSCC-derived primary cell lines, from the original cohort of 20 patients. Approximately 20 mg of tissue was homogenized using the Omni Tissue Homogenizer (Omni TH, Omni International, Kennesaw, GA, USA), before following the RNeasy Mini kit protocol (cat#74104, Qiagen, Hilden, Germany). RNA was extracted from frozen cell pellets of 5 × 10^5^ viable cells, using the RNeasy Micro kit protocol (cat#74004, Qiagen). An on-column DNase digest (cat#79254, Qiagen) step was included to remove potentially contaminating genomic DNA. RNA quantity was determined using a NanoDrop 2000 Spectrophotometer (Thermo Fisher Scientific, Waltham, MA, USA). Transcriptional expression was analyzed in triplicate using the Rotor-Gene Q (Qiagen), Rotor-Gene Multiplex RT-PCR Kit (cat#204974, Qiagen) and TaqMan Gene Expression Assay primer probes on 40 ng of RNA. The primer probes used were angiotensinogen (Hs01586213_m1), renin (Hs00982555_m1), PRR (Hs00997145_m1), ACE (Hs00174179_m1), ACE2 (Hs01085333_m1), AT_1_R (Hs00258938_m1), and AT_2_R (Hs00169126_m1; cat#4331182, Thermo Fisher Scientific). Gene expression was normalized to the reference genes GAPDH (Hs99999905_m1) and PUM1 (Hs00206469_m1; cat#4331182, Thermo Fisher Scientific). Universal human reference RNA (UHR; cat#CLT636690, Takara, Shiga, Japan)—total RNA extracted from a range of healthy adult human tissues—was used as the calibrator for the 2^∆∆Ct^ analysis. Nuclease free water was added for the no-template control. Positive controls were RNA from HepG2 cells (angiotensinogen and ACE2), PC3 cells (renin), or uterine fibroid tissue (PRR, ACE, AT_1_R, and AT_2_R). The presence of correctly sized bands from the endpoint amplification products was confirmed using 2% agarose gel electrophoresis (cat#G402002, Invitrogen) and imaged using the ChemiDoc MP (Bio-Rad, Hercules, CA, USA). Graphs were generated using GraphPad Prism (v8.0.2, San Diego, CA, USA) and results expressed as fold change relative to UHR. A fold-change cut off was set at 2.0 for upregulated, and 0.5 for downregulated, genes.

### 2.6. Western Blotting

First, 20 µg of total protein was extracted from six mHNcSCC tissue samples with snap-frozen tissue available and the four mHNcSCC-derived primary cell lines from the original cohort of 20 patients. Tissue samples macroscopically included only tumor tissue. Tissue underwent pestle homogenization (cat#PES-15-B-SI, Corning) in ice-cold Radioimmunoprecipitation assay buffer (cat#89900, Pierce Biotechnology, Rockford, IL, USA) supplemented with a protease and phosphatase inhibitor cocktail (cat#78440, Pierce Biotechnology), cell lines were extracted as above without the homogenization step. Protein was quantified using a BCA assay (cat#23227, Pierce Biotechnology), and diluted in an equal volume of 2× LDS (cat#B0007, Invitrogen). Protein was separated by SDS-PAGE and transferred to a PVDF membrane as previously described [56]. Protein was detected on the iBind Flex (cat#SLF2000, Invitrogen) using primary antibodies for angiotensinogen (1:1000; cat#79299, Cell Signaling), PRR (1:500; cat#HPA003156, Sigma-Aldrich, St. Louis, MO, USA), ACE (1:1000; cat#ab254222, Abcam), ACE2 (1:500, cat#MAB933, R&D Systems), AT_2_R (1:250; cat#ab92445, Abcam), and α-tubulin (1:2000; cat#62204, Invitrogen). Secondary antibodies used were goat antirabbit HRP (1:1000; cat#111-035-045, Jackson ImmunoResearch, West Grove, PA, USA) for angiotensinogen, PRR, ACE, and AT_2_R, goat antimouse HRP (1:1000; cat#ab6789, Abcam) for ACE2, and donkey antimouse Alexa fluor 488 (1:1000; cat#A-21202, Invitrogen) for α-tubulin. Positive controls were plasma for angiotensinogen, tonsil for PRR, mouse lung for ACE, kidney for ACE2, and mouse heart for AT_2_R. Western blotting (WB) for renin was abandoned after no antibody was found to produce a single specific band using this technique.

To visualize HRP protein bands, Clarity Western ECL substrate (cat#1705061, Bio-Rad) was used with the ChemiDoc MP Imaging System (Bio-Rad) and Image Lab 6.0 software (Bio-Rad) to analyze protein bands.

## 3. Results

### 3.1. Angiotensinogen, PRR, ACE and AT_2_R but Not Renin or ACE2 Were Present on mHNcSCC Tissue Samples

**Figure 1:**
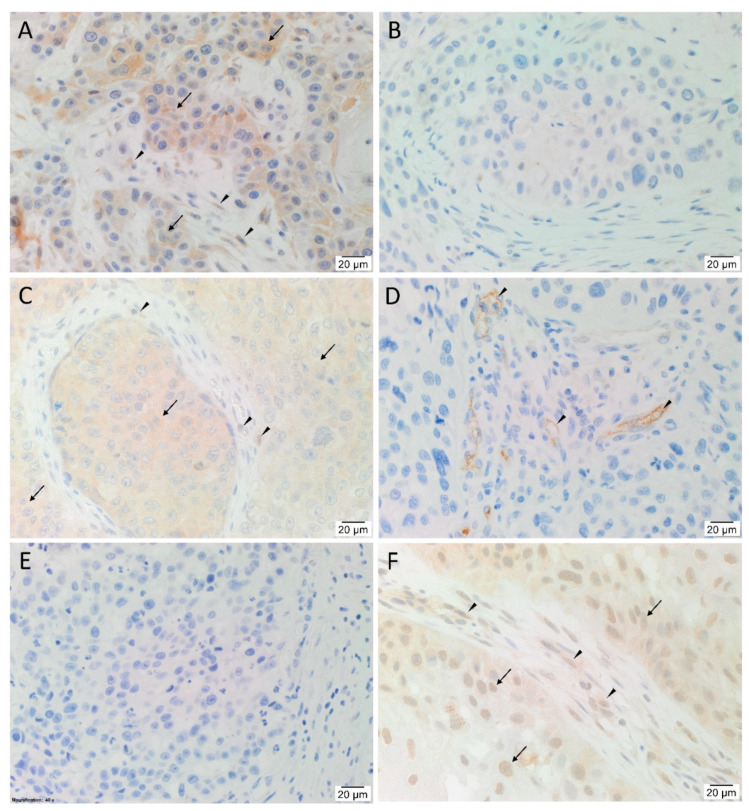
Representative immunohistochemical-stained-sections of metastatic head and neck cutaneous squamous cell carcinoma tissue samples stained for components of the renin-angiotensin system. Angiotensinogen (**A**, brown) was expressed on cells within the tumor nests (TNs, *arrows*), and to a lesser degree in the peri-tumoral stroma (PTS, *arrowheads*). Renin (**B**, brown) was not present in the tissue samples. PRR (**C**, brown), demonstrated cytoplasmic expression of cells within the TNs (*arrows*), and to a lesser extent cells within the PTS (*arrowheads*) in two cases. ACE (**D**, brown) was expressed on the endothelium of the tumor microvessels within the PTS (*arrowheads*). ACE2 (**E**, brown) was not present in the tissue samples. AT_2_R (**F**, brown) was expressed by cells within the TNs (*arrows*) and the PTS (*arrowheads*). Nuclei were counter-stained with hematoxylin (**A**–**F**, blue). Original magnification: 400×. n = 20.

H&E staining (Figure S1A) confirmed the presence of mHNcSCC organized into tumor nests (TNs) with intervening peritumoral stroma (PTS) in all 20 tissue samples. Immunohistochemical staining showed heterogenous expression of angiotensinogen (Figure 1A), with 12 samples showing mostly weak cytoplasmic staining, four samples with weak nuclear staining in TNs, and five samples with cytoplasmic staining of cells within the PTS. There was no expression of renin (Figure 1B) in any of the tissue samples. PRR (Figure 1C) showed cytoplasmic expression on the cells within the TNs of all samples, with minimal staining of the PTS in two cases. Membranous expression of ACE (Figure 1D, brown) was present on the endothelium of the tumor microvessels within the PTS in all samples. ACE2 (Figure 1E, brown) was not detected in any of the samples. AT_2_R (Figure 1F) was observed in 19 of the 20 samples with weak to moderate cytoplasmic staining of cells within the TNs, and to a lesser degree the PTS. Nuclear staining of AT_2_R was present in 14 cases, mostly in the TNs with some staining in the PTS.

Human tissues used for positive controls for immunohistochemical staining showed the expected staining pattern for angiotensinogen (Figure S1B, brown) in liver, renin (Figure S1C, brown) in kidney, PRR (Figure S1D, brown) in placenta, and ACE (Figure S1E, brown), ACE2 (Figure S1F, brown), and AT_2_R (Figure S1G, brown) in kidney. mHNcSCC samples with the relevant isotype negative controls demonstrated no staining (Figure S1H, brown). Tissue negative controls showed appropriate negative staining and confirmed the specificity of the primary antibodies (Figure S2A–F, brown).

### 3.2. Angiotensinogen, PRR, and AT_2_R Were Expressed by the CSCs in the TNs and PTS, and ACE Was Expressed by the Endothelium of the Tumor Microvessels within mHNcSCC Tissue Samples

**Figure 2:**
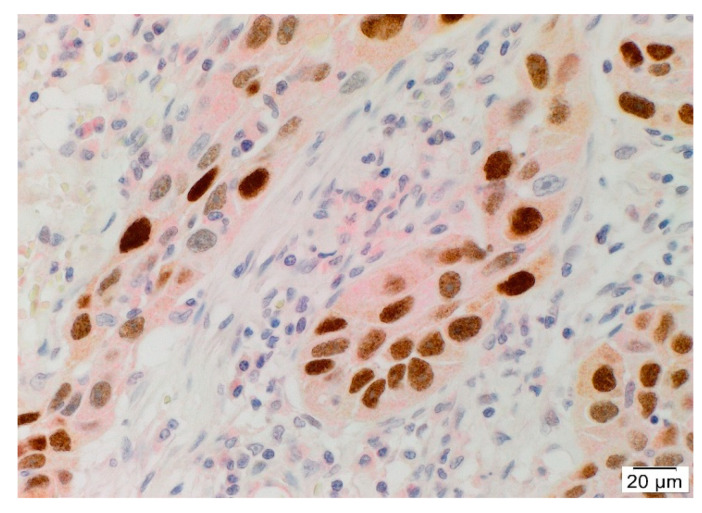
Representative double immunohistochemical-stained section of metastatic head and neck cutaneous squamous cell carcinoma demonstrating cytoplasmic expression of angiotensinogen (red) in CSCs with nuclear expression of SOX2 (brown), within the TNs. Cell nuclei were counter-stained with hematoxylin (blue). Original magnification: 400×. n = 2.

Double immunohistochemical staining of angiotensinogen and SOX2 demonstrated expression of angiotensinogen (Figure 2, red) by SOX2+ (Figure 2, brown) CSCs within the TNs. Positive controls for double immunohistochemical staining showed appropriate cytoplasmic staining in the liver for angiotensinogen (Figure S3A), and squamous epithelium of the tonsil for SOX2 (Figure S3B), with no staining on the isotype negative controls (Figure S3C,D).

**Figure 3:**
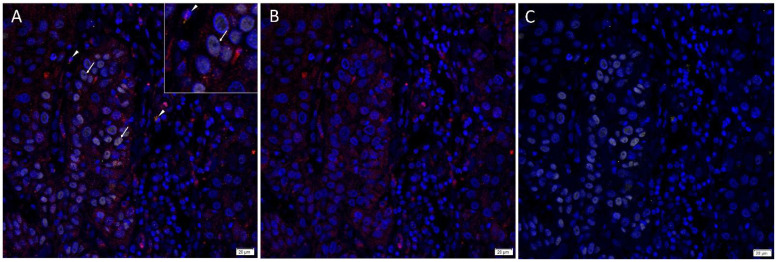
Representative merged and split immunofluorescence-stained sections of metastatic head and neck cutaneous squamous cell carcinoma demonstrating the expression of PRR (**A**,**B**, red) by SOX2+ (**A**,**C**, yellow) CSCs throughout the tumor nests (TNs, *arrows*) and the peritumoral stroma (PTS, *arrowheads*). Cell nuclei were counter-stained with 4′,6-diamidino-2-phenylindole (**A**–**C**, blue). The inserts show enlarged views of the corresponding images Original magnification 400×. n = 2.

**Figure 4:**
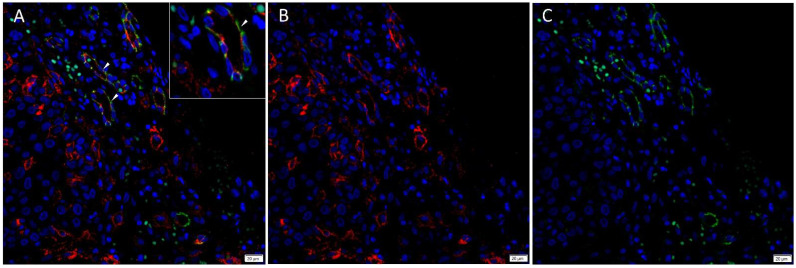
Representative merged and split immunofluorescence-stained sections of metastatic head and neck cutaneous squamous cell carcinoma demonstrating the expression of ACE (**A**,**B**, red) by the CD31+ (**A**,**C**, green) endothelium (*arrowheads*) of the tumor microvessels within the PTS. Cell nuclei were counter-stained with 4′6-diamidino-2-phenylindole (**A**–**C**, blue). The inserts show enlarged views of the corresponding images Original magnification 400×. n = 2.

**Figure 5:**
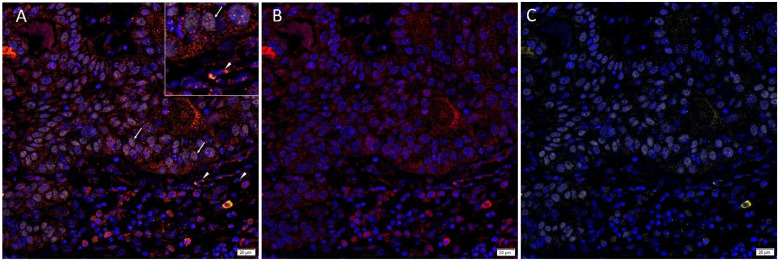
Representative merged and split immunofluorescence-stained sections of metastatic head and neck cutaneous squamous cell carcinoma demonstrating the expression of AT_2_R (**A**,**B**, red) in the cytoplasm and nucleus of the SOX2+ (**A**,**C**, yellow) CSCs within the TNs (*arrows*) and the PTS (*arrowheads*). Cell nuclei were counter-stained with 4′6-diamidino-2-phenylindole (**A**–**C**, blue). The inserts show enlarged views of the corresponding images Original magnification 400×. n = 2.

Immunofluorescence staining demonstrated expression of PRR (Figure 3A,B, red) by the SOX2^+^ (Figure 3A,C, yellow) CSCs throughout the TNs (*arrows*) and to a lesser extent the PTS (*arrowheads*). ACE (Figure 4A,B, red) was localized to the CD31+ (Figure 4A,C, green) endothelium of the tumor microvessels within the PTS. AT_2_R (Figure 5A,B, red) was expressed in the nucleus of the SOX2+ (Figure 5A,C, yellow) CSCs within the TNs (*arrows*) and in some cells within the PTS (*arrowheads*). Figure inserts have been provided to show enlarged views of the corresponding images.

Specificity of the secondary antibodies was confirmed in the isotype negative control (Figure S4), which demonstrated minimal staining.

### 3.3. RT-qPCR Demonstrated Transcript Expression of Angiotensinogen, PRR, ACE, ACE2, and AT_1_R, but Not Renin or AT_2_R

**Figure 6:**
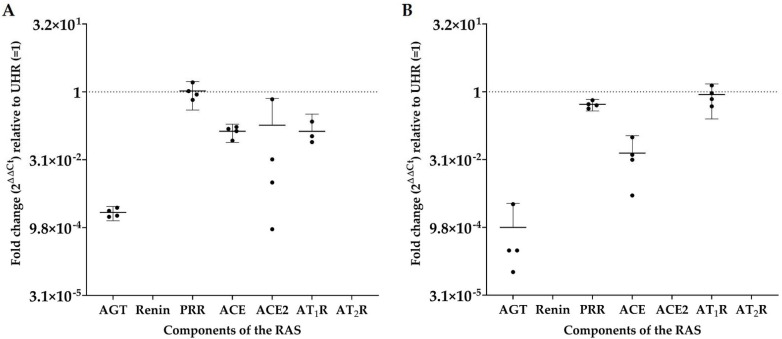
Fold-change (2^ΔΔCt^) in transcript expression of components of the renin-angiotensin system (RAS): angiotensinogen (AGT), renin, PRR, ACE, ACE2, AT_1_R, and AT_2_R, as determined by RT-qPCR in four metastatic head and neck cutaneous squamous cell carcinoma (mHNcSCC) tissue samples (**A**) and four mHNcSCC-derived primary cell lines (**B**). CT values were normalized to the reference genes GAPDH and PUM1 to calculate ΔCT, and expression compared to that of UHR (y = 1). Error bars represent 95% confidence intervals of the mean. Graphs are shown with log2 scale.

Angiotensinogen, PRR, ACE, ACE2, and AT_1_R were detected in all four mHNcSCC tissue samples, while renin and AT_2_R were not detected in any of the four samples; see Figure 6A. PRR expression was similar to that of UHR, with downregulated relative expression of angiotensinogen, ACE, ACE2, and AT_1_R. Angiotensinogen, PRR, ACE, and AT_1_R were expressed in all four mHNcSCC-derived primary cell lines, while renin, ACE2, and AT_2_R were not detected (Figure 6B). Expression of AT_1_R and PRR was similar to that of UHR, while angiotensinogen and ACE were downregulated relative to UHR. The expected size amplicons were observed for tissue samples (Figure S5) and cell lines (Figure S6), with no products observed in the no-template control reactions.

### 3.4. Western Blotting Demonstrated Protein Expression of Angiotensinogen, PRR, and ACE

**Figure 7:**
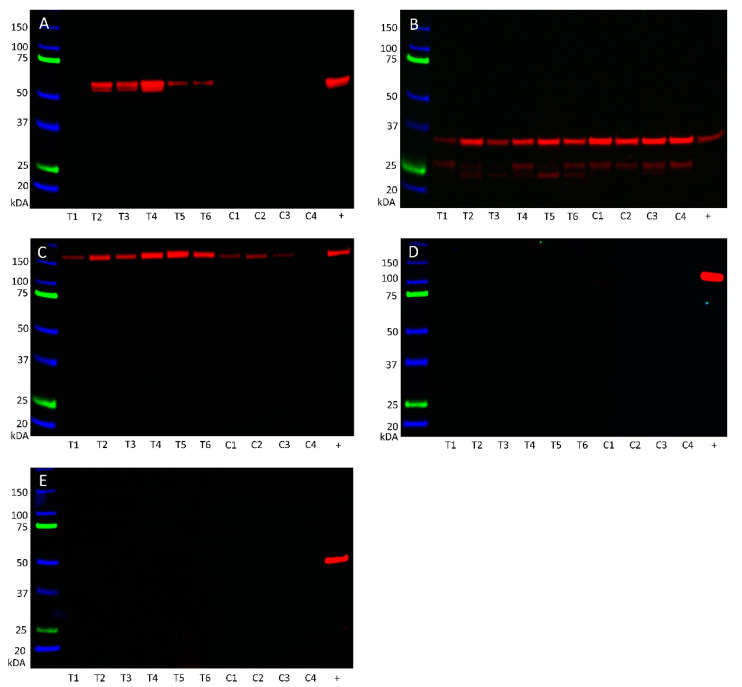
Western blotting of protein extracted from six metastatic head and neck cutaneous squamous cell carcinoma (mHNcSCC) tissue samples (T1–T6) and four mHNcSCC-derived primary cell lines (C1–C4) detected the expression angiotensinogen (**A**) in five of the six tissue samples at the appropriate molecular weight of 55 kDa, but not in any of the mHNcSCC-derived primary cell lines. PRR (**B**) was detected in all of the six tissue samples and four primary cell lines at the expected molecular weight at 35 kDa for the full-length transmembrane isoform and the shorter secreted isoform. ACE (**C**) was detected in all 6 tissue samples, but only three of the four cell lines, at the appropriate weight of 195 kDa. ACE2 (**D**) and AT_2_R (**E**) were not detected in any of the six tissue samples or four primary cell line samples investigated, but were present in the positive controls. Lanes 1–6 indicate six tissue samples used, lanes 7–10 indicate cell lines. +ve indicates positive control: plasma for angiotensinogen; tonsil for PRR; mouse lung for ACE; kidney for ACE2; and mouse heart for AT_2_R. The molecular weight ladder (kDa) is labeled for each blot. Blots for α-tubulin are presented in Figure S7.

Angiotensinogen (Figure 7A) was observed at the appropriate size of 55 kDa in five of the six mHNcSCC tissue samples, but was not detected in any of the four mHNcSCC-derived primary cells lines. PRR (Figure 7B) was detected at the appropriate size for the transmembrane isoform at 35 kDa in all of the six tissue samples and four primary cell lines, with all samples also showing the shorter secreted isoform at 28 kDa. ACE (Figure 7C) was demonstrated in all six mHNcSCC tissue samples, and three of the four primary cell lines at the expected size of 195 kDa. ACE2 (Figure 7D) was not detected in any of the six tissue samples or the four primary cell lines. AT_2_R (Figure 7E) was below detectable levels in all six tissue samples and four primary cell lines, with the positive control showing a band at the expected size of 40 kDa in mouse heart. WB of α-tubulin (Figure S7) confirmed approximately equal protein loading for all samples.

## 4. Discussion

There is increasing evidence of the role of the RAS in tumorigenesis, and metastasis [23,24,57]. We have recently demonstrated the presence of CSC subpopulations that express the ESC markers OCT4, NANOG, SOX2, KLF4, and c-MYC in primary HNcSCC [19] and mHNcSCC [20]. In this study we have shown expression of five components of the RAS: angiotensinogen, PRR, ACE, AT_1_R, and AT_2_R, in mHNcSCC tissue samples.

Angiotensinogen expression was demonstrated in the cytoplasm of cells within the TNs by immunohistochemical staining, with protein and transcript expression confirmed by WB and RT-qPCR respectively. Protein expression of angiotensinogen was not detected by WB in the mHNcSCC-derived primary cell lines, which may signify that expression is dependent on the extracellular environment, which is lost during the culturing process. Double immunohistochemical staining demonstrated localization of angiotensinogen to the SOX2+ CSCs within the TNs. Zhang et al. [58] have also identified angiotensinogen as a key gene in tongue SCC using a bioinformatics approach. Although the role of angiotensinogen in carcinogenesis has yet to be elucidated, it is an important precursor in the RAS cascade [24,57].

PRR was demonstrated on the SOX2+ CSCs within the TNs, and, to a lesser extent, within the PTS by immunohistochemical and immunofluorescence staining. RT-qPCR confirmed transcript expression and WB confirmed protein expression of PRR in the mHNcSCC tissue samples and mHNcSCC-derived primary cell lines. PRR has been previously associated with CSC proliferation, via the Wnt/β-catenin signaling [59,60], and is implicated in carcinogenesis [24,60]. Aberrant PRR expression has also been shown to occur during the early stages of carcinogenesis, such as in pancreatic ductal carcinoma [60].

Although renin was not detected by the techniques used in this study, this does not preclude the local conversion of angiotensinogen to ATI, as there are known bypass loops of the RAS [23,24,57]. For example, cathepsin D converts angiotensinogen to ATI, bypassing the action of renin [61,62]. Cathepsin D is known to be present in SCC [63,64] and has been associated with tumor invasion, poor prognosis, and nuclear accumulation of p53 protein in esophageal SCC [65]. Future investigation could explore the expression and function of cathepsin D and other bypass loops of the RAS in mHNcSCC.

ACE was demonstrated on the endothelium of the tumor microvessels within the PTS by immunohistochemical and immunofluorescence staining, with expression confirmed by WB and RT-qPCR. The localization of ACE to the endothelium of the tumor microvessels suggests a role in vasculogenesis, which may reflect ‘vascular mimicry’ [66] and ACE has been identified as a critical regulator of hemangioblast differentiation [28]. In addition, ACE may regulate EMT [67], a central aspect of cancer progression and an essential part of metastasis [66]. There are known bypass loops of ACE, specifically chymase, which converts ATI to ATII [68]. Chymase has also been observed in gastric [69], lung [70], and uterine cervical [71] cancers. Chymase+ mast cells have also been proposed to play a role in SCC [72,73,74], possibly by promoting angiogenesis [75]. Interestingly, SCC antigen, a serological marker of SCC tumor advancement, has been demonstrated to inhibit chymase and cathepsin G, another bypass of the RAS [76].

ACE2 was not found in any of the mHNcSCC tissue samples investigated. ACE2 has been shown to reduce lung cancer metastasis in a mouse model by inhibiting EMT [77]. Another study investigated the tumor-suppressive mechanisms of ACE2 via angiogenesis and tumor invasion, and found these to be mediated by regulation of MMP-2, MMP-9, and VEGFa [78]. Similar antitumor effects have been described in gallbladder [79], pancreatic [80], breast [81,82], and other cancer types [42]. An alternative pan-cancer approach using the Cancer Genome Atlas has also identified ACE2 as a protective factor, with improved immunotherapy response and impaired tumor progression [83]. Pathways identified included cell cycle activity, mismatch repair, and TGF-β, Wnt, VEGF, and NOTCH signaling pathways [83], which are also proposed therapeutic targets for CSCs [84]. The absence of ACE2 in our samples is therefore a significant finding, suggesting increased signaling down these pathways.

AT_1_R blockers reduce tumor size and vascularization [44], with an observed improved prognosis in esophageal SCC [85]. AT_1_R may also contribute to carcinogenesis through angiogenesis, as it promotes VEGF signaling [43]. Interestingly, differential signaling through AT_1_R and AT_2_R has been shown to alter the differentiation of hemangioblasts [28]. Given that ATII has also been shown to stimulate the proliferation of early progenitor cells through a receptor mediated response [86], this suggests the RAS may have a yet unknown role in differentiation. AT_1_R mRNA was detected in all four mHNcSCC tissue samples, which is consistent with our previous finding of AT_1_R overexpression in cSCC [44]. Unfortunately we were unable to validate a suitable antibody for AT_1_R [52,53,54,55], thus precluding this marker from protein-level analysis in this study.

The observation of nuclear expression of AT_2_R by both immunohistochemical and immunofluorescence staining in our samples is interesting, given that it is classically a transmembrane receptor [87]. Nonetheless, this finding is consistent with other reports demonstrating the presence of both AT_1_R and AT_2_R in the nuclei of cardiac fibroblasts [88], and AT_1_R in the nuclei of hepatocytes [89]. ATII receptors may regulate nuclear roles for ATII, such as fibroblast proliferation, collagen expression, RNA synthesis and nitric oxide regulation [88,90]. It is also intriguing that AT_2_R was detected by immunohistochemical and immunofluorescence staining of mHNcSCC tissue samples, but not by WB and RT-qPCR. Possible reasons for this include the possibility of the presence of an mRNA splice variant or polymorphism that was not detected by RT-qPCR [91], protein levels being below detectable levels by WB, or nonspecific binding of the antibody used in immunohistochemical and immunofluorescence staining. The former is consistent with previous findings in glioblastoma [8]. It is also possible that the lack of detectable AT_2_R in the mHNcSCC tissue samples and mHNcSCC-derived primary cell lines by RT-qPCR, may be due to redundancy of AT_2_R [51]. Given conflicting evidence suggesting both a pathological and a protective role for AT_2_R [23,90], AT_2_R isoforms and their contribution in mHNcSCC remain a topic for further investigation.

In this study, components of the RAS were not demonstrated in some of the tissue samples by WB and RT-qPCR analyses, despite their identification by immunohistochemical and immunofluorescence staining. This may be due to sample degradation and variability between tissue samples and a larger sample size may minimize these limitations. Further work with functional experiments is needed to elucidate the precise regulatory mechanisms of the RAS on the CSCs within mHNcSCC.

Our novel findings of CSCs in mHNcSCC expressing components of the RAS are consistent with findings of previous studies of OCSCC of different subsites [47,48], glioblastoma [8], metastatic melanoma to the brain [18] and the regional nodes [49], metastatic colon adenocarcinoma [50], and primary HNcSCC [51]. This may explain the observed reduced risk of developing cSCC in patients who are administered ACE inhibitors and AT_1_R blockers [20,27]. It also suggests that CSCs within mHNcSCC may be a novel therapeutic target through modulation of the RAS, using commonly available medications, in the treatment of this aggressive cancer [92,93].

## Data Availability

The data presented in this study are available on request from the corresponding author.

## Supplementary Materials

The following are available online at https://www.mdpi.com/2073-4409/10/2/243/s1, Table S1: Demographic details of patients and their tumors, Figure S1: H&E and immunohistochemical positive controls, Figure S2: Tissue negative controls, Figure S3: Double immunohistochemical staining split individual images and positive controls, Figure S4: Immunofluorescence isotype control, Figure S5: RT-qPCR electrophoresis of tissue samples, Figure S6: RT-qPCR electrophoresis of cell line samples, Figure S7: Western blot of α-tubulin loading control.
