# Supplementary material for: Cancer Stem Cells in Metastatic Head and Neck Cutaneous Squamous Cell Carcinoma Express Components of the Renin-Angiotensin System
Source: Cells. 2021 Jan 27;10(2):243. doi: 10.3390/cells10020243 (PMC7910940; doi:10.3390/cells10020243)
Supplement: Supplementary file 1 [file cells-10-00243-s001.pdf]

Supplementary Materials

Table S1. Patient demographics and site of nodal metastasis of metastatic head and neck cutaneous squamous cell carcinoma.

Patient	Sex	Age (years)	Anatomic site
1	M	51	Neck
2	M	59	Neck
3	M	73	Neck
4	M	74	Parotid and neck
5	M	74	Parotid
6	M	75	Parotid
7	M	76	Parotid
8	F	77	Parotid
9	M	77	Parotid
10	M	78	Parotid
11	M	79	Parotid
12	M	80	Parotid
13	M	82	Parotid
14	M	83	Parotid
15	M	85	Neck
16	F	85	Parotid
17	M	86	Parotid
18	M	86	Neck
19	M	86	Parotid
20	M	87	Parotid

M, male; F, female.

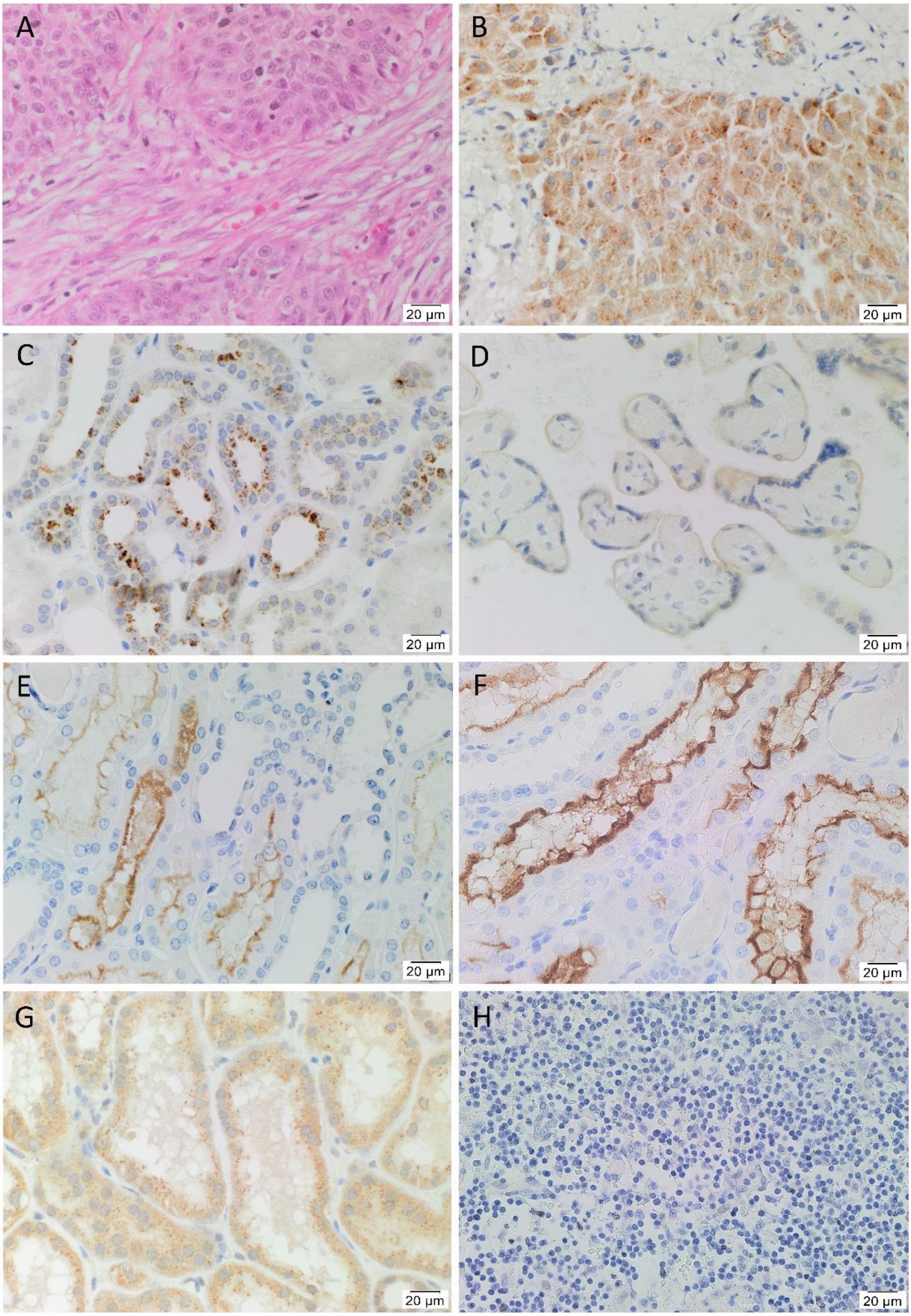

Figure S1. Representative hematoxylin and eosin stained slides of metastatic head and neck cutaneous squamous cell carcinoma (mHNcSCC) tissue samples demonstrating the tumor nests surrounded by peritumoral stroma (A). Positive human control tissues for immunohistochemical staining for angiotensinogen (B, brown) in liver, renin (C, brown) in kidney, PRR (D, brown) in placenta, and ACE (E, brown), ACE2 (F, brown), and AT_2R (G, brown) in kidney. A mHNcSCC section

was used as an isotype negative control for immunohistochemical staining (H). Nuclei were counter-stained with hematoxylin (B-H, blue). Original magnification 400x.

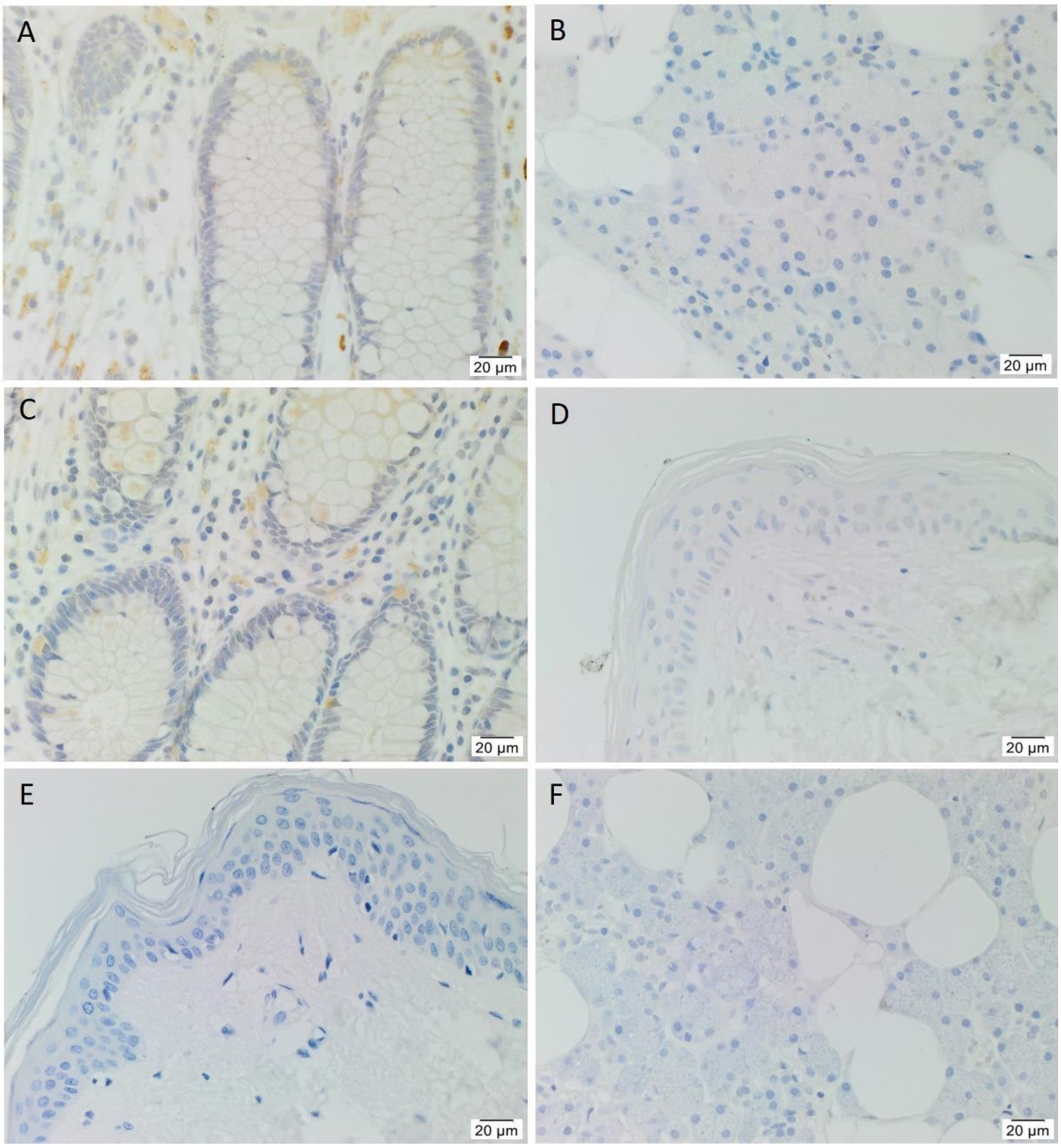

Figure S2. Tissue negative control samples confirming specificity of the primary antibodies. Tissue negative normal human control tissues for immunohistochemical staining for angiotensinogen (A, brown) in colon, renin (B, brown) in salivary gland, PRR (C, brown) in colon, ACE (D, brown) and ACE2 (E, brown) in skin, and AT_2R (F, brown) in salivary gland showed appropriately negative staining. Nuclei were counter-stained with hematoxylin (A-F, blue). Original magnification 400x.

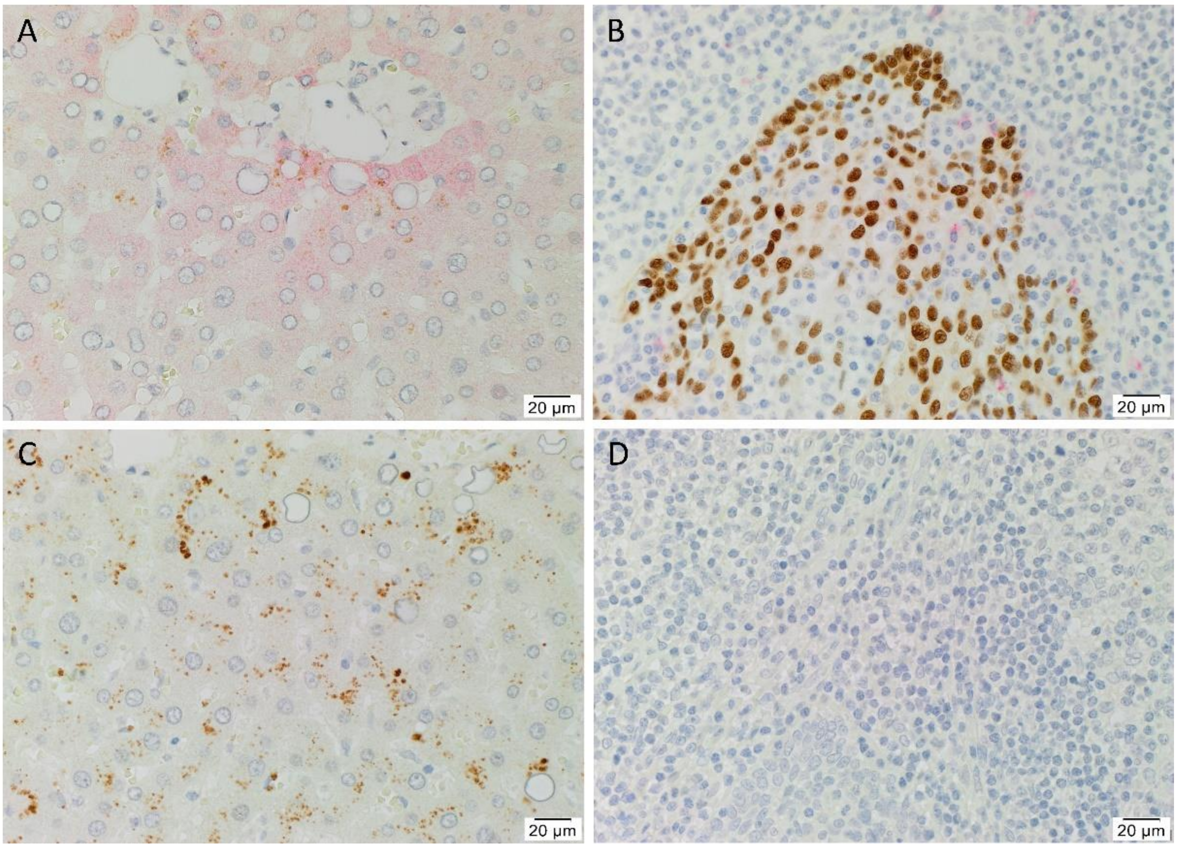

Figure S3. Positive controls for double immunohistochemical staining for angiotensinogen (A, red) in liver, and SOX2 (B, brown) in tonsillar epithelium. Isotype negative controls showed no staining for angiotensinogen (C, red) in liver, or SOX2 (D, brown) in tonsillar epithelium. Note that the brown staining seen in panel C is lipofuscin, as confirmed by an anatomical pathologist. Cell nuclei were counter-stained with hematoxylin (A-D, blue). Original magnification: 400x.

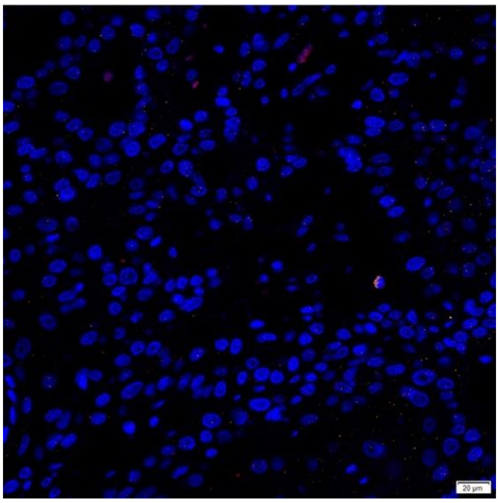

Figure S4. A representative isotype negative control on a section of mHNcSCC. Cell nuclei were counter-Scheme 4. diamidino-2-phenylindole (blue). Original magnification: 400x.

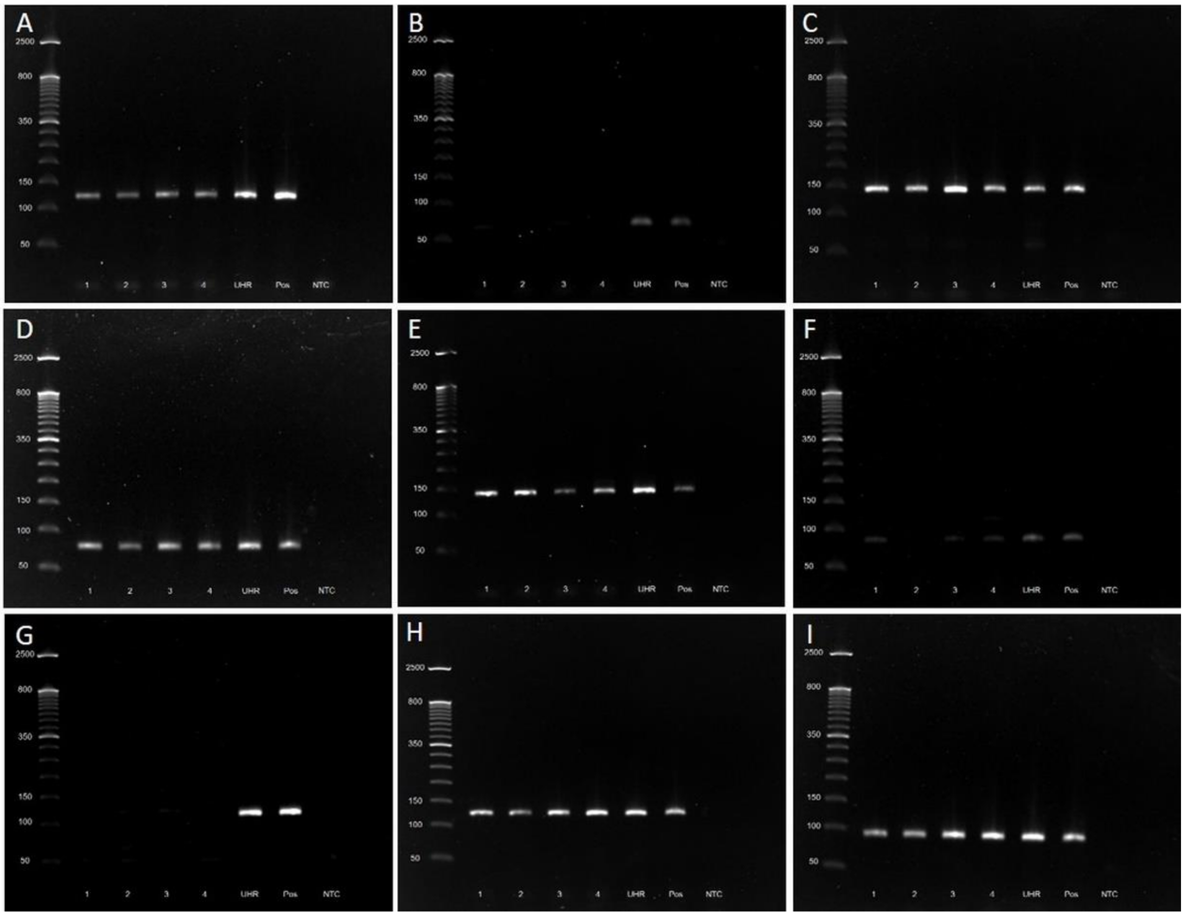

Figure S5. RT-qPCR amplification products from four metastatic head and neck cutaneous squamous cell carcinoma tissue samples (A-I) were checked using agarose gel electrophoresis to confirm probe specificity. Only the expected amplicon for each TaqMan assay was observed for angiotensinogen (A, 121bp), renin (B, 62bp), PRR (C, 141bp), ACE (D, 74bp), ACE2 (E, 141bp), AT_1R (F, 80bp), AT_2R (G, 113bp), GAPDH (H, 122bp), and PUM1 (I, 89bp). Ladder refers to the DNA molecular weight marker in base pairs (bp); Lanes 1-4 refer to the respective tissue samples; Pos, positive control (HepG2 cells for angiotensinogen and ACE2, PC3 cells for renin, and uterine fibroid tissue for PRR, ACE, AT_1R and AT_2R); NTC, no template control (RNase-free water) to confirm no contamination.

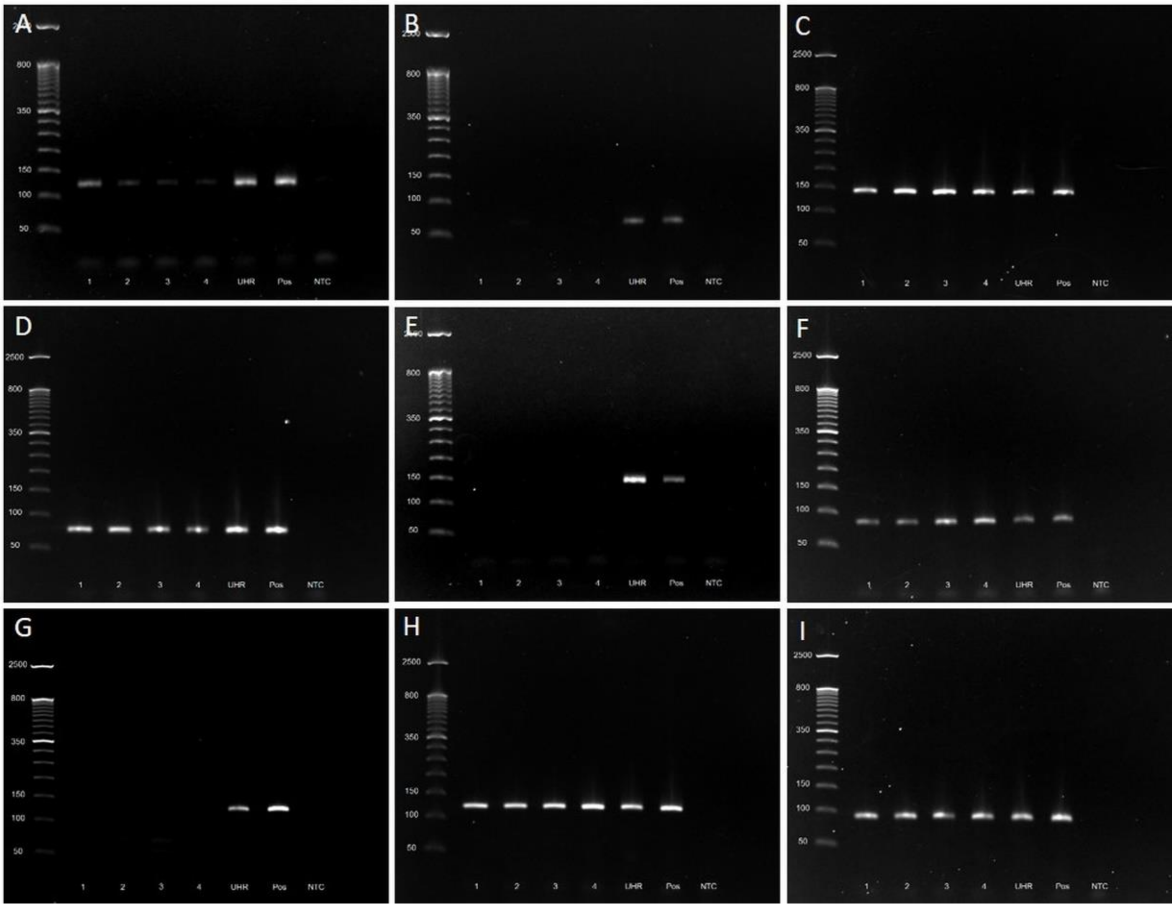

Figure S6. RT-qPCR amplification products from four metastatic head and neck cutaneous squamous cell carcinoma-derived primary cell line samples (A-I) were checked using agarose gel electrophoresis to confirm probe specificity. Only the expected amplicon for each TaqMan assay was observed for angiotensinogen (A, 121bp), renin (B, 62bp), PRR (C, 141bp), ACE (D, 74bp), ACE2 (E, 141bp), AT_1R (F, 80bp), AT_2R (G, 113bp), GAPDH (H, 122bp), and PUM1 (I, 89bp). Ladder refers to the DNA molecular weight marker in base pairs (bp); Lanes 1-4 refer to the respective cell line samples; Pos, positive control (HepG2 cells for angiotensinogen and ACE2, PC3 cells for renin, and uterine fibroid tissue for PRR, ACE, AT_1R and AT_2R); NTC, no template control (RNase-free water) to confirm no contamination.

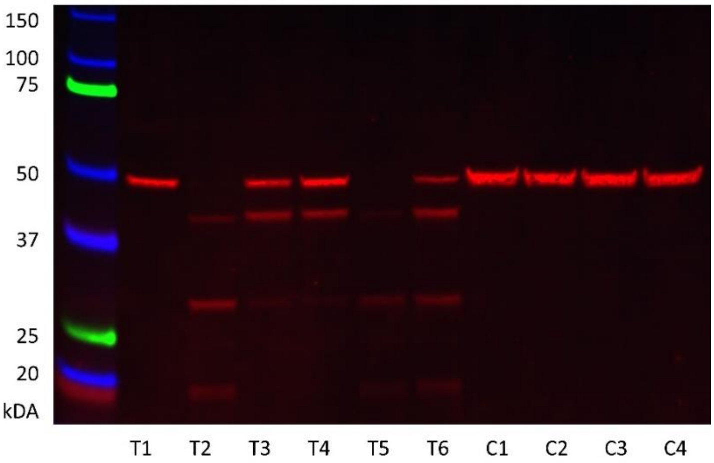

Figure S7. Western blotting of protein extracted from metastatic head and neck cutaneous Scheme 1. indicate 6 tissue samples, 7-10 indicate cell lines. The molecular weight ladder (kDa) is labeled.
